# CNS Organoid Surpasses Cell-Laden Microgel Assembly to Promote Spinal Cord Injury Repair

**DOI:** 10.34133/2022/9832128

**Published:** 2022-08-03

**Authors:** Zitian Wang, Haoran Zhao, Xiaowei Tang, Tianyu Meng, Davit Khutsishvili, Bing Xu, Shaohua Ma

**Affiliations:** ^1^Tsinghua Shenzhen International Graduate School, Tsinghua University, Shenzhen 518055, China; ^2^Tsinghua-Berkeley Shenzhen Institute, Shenzhen 518055, China; ^3^Shenzhen Bay Laboratory, Shenzhen, China; ^4^Institute for Brain and Cognitive Sciences, Tsinghua University, Beijing 100084, China

## Abstract

The choice of therapeutic agents remains an unsolved issue in the repair of spinal cord injury. In this work, various agents and configurations were investigated and compared for their performance in promoting nerve regeneration, including bead assembly and bulk gel of collagen and Matrigel, under acellular and cell-laden conditions, and cerebral organoid (CO) as the *in vitro* preorganized agent. First, in Matrigel-based agents and the CO transplantations, the recipient animal gained more axon regeneration and the higher Basso, Beattie, and Bresnahan (BBB) scoring than the grafted collagen gels. Second, new nerves more uniformly infiltrated into the transplants in bead form assembly than the molded chunks. Third, the materials loaded the neural progenitor cells (NPCs) or the CO implantation groups received more regenerated nerve fibers than their acellular counterparts, suggesting the necessity to transplant exogenous cells for large trauma (e.g., a 5 mm long spinal cord transect). In addition, the activated microglial cells might benefit from neural regeneration after receiving CO transplantation in the recipient animals. The organoid augmentation may suggest that *in vitro* maturation of a microtissue complex is necessary before transplantation and proposes organoids as the premium therapeutic agents for nerve regeneration.

## 1. Introduction

Extensive biomaterials have long been developed and widely studied in regenerative medicine [1], but the choice of therapeutic agents remains unclear and lacks standards for large-scale tissue repair. Engineered biomaterials, designed to take advantage of characteristics or the bioactive components they load, are aimed at facilitating the in situ tissue regeneration by reducing inflammation or attracting the host restorative cells to repair the damage after transplantation. Without arousing the associated rejection reaction and ethical issues, the biomaterials enable the tissue at the injury site as a bioreactor to achieve effective in situ regeneration and self-healing instead of bringing in allogeneic and xenogeneic cells [2–4]. Yet with merits also come limitations. Material-only administration is proven not universally applicable because it relies on the type of target tissue and age of recipients [5, 6].

Because traumatic spinal cord injury (SCI) exhibits multifaceted and complex pathological features [7] and the central nervous system (CNS) has limited regenerative capacity, the repair process and the standards of choice for therapeutic agents are complicated and more apprehensive. Transplantation of liquid and bulk molded scaffolds [8, 9], as well as scaffold conduits [10–14], received partial functional recovery in laboratory and clinical trials of SCI repair. In recent years, bead assembly of scaffolding materials received hospitality because of the reduced transplantation invasiveness, increased volume penetration for nutrients and host cells, wide adaptability of the assembled block toward trauma volume, and increased pliability to shear [15–19].

Numerous studies using neural progenitor cells (NPCs) as the therapeutic agents have been reported in the SCI treatment [20–22]. Neurogenesis benefits from various secreted growth factors from the exogenous stem cells [23, 24]. However, stem cell transplantation is proven not satisfactory because of the short transport span of delivered cells and low rates of stem cell homing [25, 26]. Yet the transplantation of stem cell and scaffold complex is expected to improve the therapy outcome because the biophysical and chemical properties render the scaffold to provide suitable environmental cues—increasing the survival rates of transplanted cells [27, 28]. Further, stem cell-laden scaffolds may compensate for the insufficiency of resident cell infiltration in large-scale trauma when acellular scaffolds are filling the cavity [29, 30].

On the other hand, to diminish the lack of effective cell interactions and unsynchronized organization and differentiation of transplanted exogenous cells with resident cells, *in vitro* developed CNS organoids derived from pluripotent stem cells (PSCs) were investigated after transplantation. The organoids possess the structural, morphological, and electrophysiological properties of the mature tissues of CNS *in vitro* [31–36]. After *in vivo* graft, the CNS organoid merged with host tissue and became functionally connected and vascularized [32, 37].

**Figure 1:**
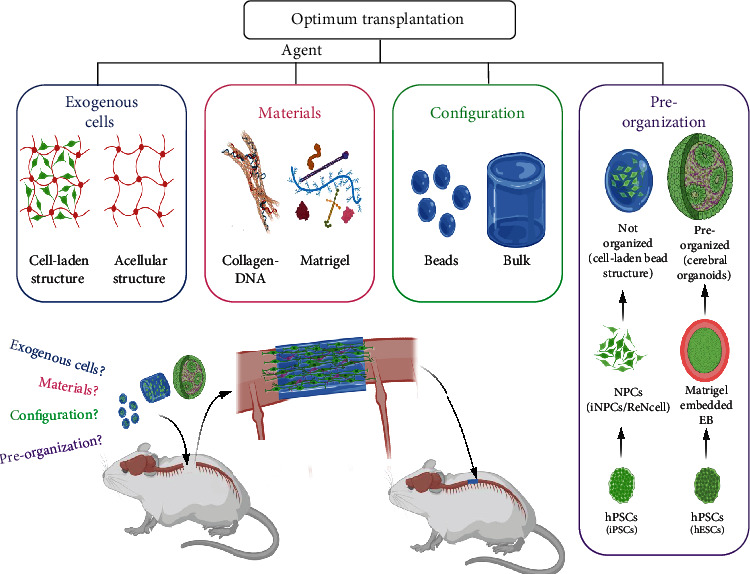
Schematic illustration of the group setting and the materials used in this study. Briefly, the biomaterials, collagen-DNA and Matrigel, were compared with the configurations of beads and bulk and with exogenous NPCs loaded or not in the transplantation of SCI. Moreover, the exogenous cells, preorganized (i.e., cerebral organoids) or unorganized (i.e., cell-laden hydrogel), were compared as well in this study.

It is a topic of debate to decide the most optimal therapeutic agent among the choices of stem cells, scaffolds, cell-scaffold complex, and their configurations. A further argument emerges with the rapid development of organoid technology, that is, the choice between unreacted stem cell-scaffold complex and the *in vitro*-reacted (matured) organoid. There has been no report on a systematic comparison of different agents for SCI repair. Herein, the parallel posttransplantation outcome was compared with collagen [16, 38, 39] and Matrigel [40, 41] as the scaffolding materials; bead assembly and molded bulk gel as the administration configuration; and cell-laden complex and materials only and cell-laden beads and CNS organoids as the reaction condition. The comparison was performed in the rat transected SCI models and characterized by histological assessments and locomotor functional recovery (Figure 1).

## 2. Results

### 2.1. Transplantation Agents: Cell-Laden Collagen Beads, Acellular Collagen Beads, and Bulk Collagen

**Figure 2:**
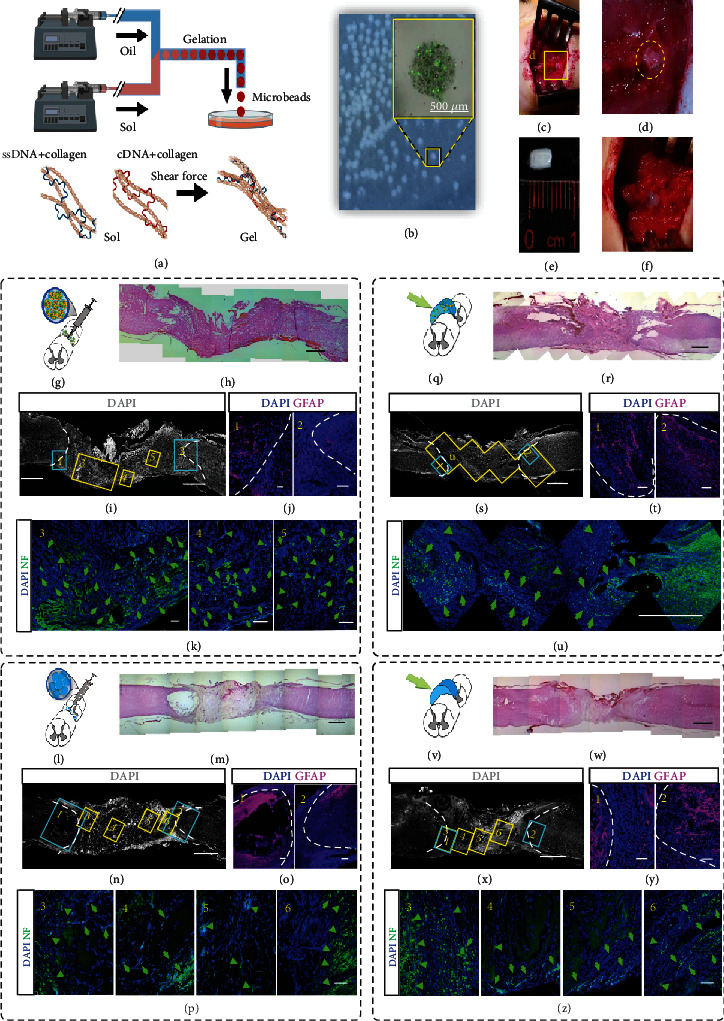
Implantation of collagen-DNA (col-DNA) gel beads and bulk gel to intertransects of spinal cords. (a) Schematic of the production of col-DNA gel beads using the cascade tubing microfluidic (CTM) technique. The gel rapid-crosslinking is induced by electrostatic attraction between collagen and DNA and mechanical interlock formation from complementary ssDNA under shear conditions. (b) The col-DNA beads in PBS. The zoom-in picture shows the overlap of fluorescence and bright-field images of a bead using calcein-AM/PI staining. (c) The cell-laden or acellular col-DNA beads are injected into the intertransects of a rat spinal cord. (d) Enlarged view of the implanted beads between the transects. (e) A molded piece of cell-laden or acellular col-DNA bulk gel matching the volume of the intertransects of a rat spinal cord. (f) Enlarged view of the implanted gel. (g) Sketch illumination of cell-laden col-DNA bead injection into the intertransects of a spinal cord. Each bead is ~500 *μ*m in diameter. (h) H&E staining of the transected spinal cord at 8 weeks after receiving bead implantation. (i–k) Confocal sagittal images show the axon regeneration (neural filament, NF) and astrocyte accumulation (GFAP) within the implants (j). Areas 1-5 are enlarged in (j) and (k). The GFAP signals in 1 and 2, artificially labeled in magenta, indicate the astrocytes at the rostral and caudal boundaries (white dot line) at 8 weeks postimplantation (j). The green arrows indicate the signals of regenerated axons by staining NF (k). (l–p) The sketch and imaging illustrations of acellular col-DNA bead (~500 *μ*m in diameter) implantation. (q) Sketch illumination of cell-laden col-DNA gel implanted to the intertransects of a spinal cord. Each gel has the size of ~5 mm (axial length) × 3 mm (transversal diameter). (r–u) H&E staining (r) and confocal sagittal imaging show axon regeneration (neural filament (NF)) and astrocyte accumulation (GFAP) within the implants (s–u). (v–z) The sketch and imaging illustrations of acellular col-DNA gel implantation. Scale bars, 100 *μ*m (j, k, o, p, t, y, z), 500 *μ*m (b), and 1 mm (h, i, m, n, r, s, u, w, x).

Collagen has been a prevalent material in engineering artificial tissues and promoting tissue healing [42, 43]. To investigate the capability of collagen and its configuration in promoting neural regeneration, we first synthesized the cell-laden collagen-DNA (col-DNA or collagen) microbeads by using the cascade-tubing microfluidic (CTM) technique [44, 45] (Figures 2(a) and 2(b)). Ultralong ssDNA (0.2 mg/mL), synthesized by rolling circle amplification (RCA), was incorporated into the collagen (2 mg/mL) scaffold to induce rapid crosslinking at physiological conditions [44]. The NPCs were generated from the human-induced pluripotent stem cells (hiPSCs) (Figure S2) and encapsulated into the microbeads. The cell-laden collagen beads or acellular col-DNA beads (Figures 2(c) and 2(d)) and the cell-laden bulk collagen or acellular bulk collagen (Figures 2(e) and 2(f)) were transplanted into the cavity where the rat spinal cord tissue of 4~5 mm in length was removed (Figure S4). Eight weeks after transplantation, the spinal cord tissues receiving cell-laden collagen bead injection were harvested and cryosectioned subsequently. Hematoxylin and eosin (H&E) staining showed that the grafted microgels had fused with the host tissues and regenerated collagen implant-derived neural tissues (Figures 2(h) and 2(i)). The immunostaining of axon marker neurofilament (NF, heavy) revealed scattered signals in the grafts (Figure 2(k)). The astrocytes were marked by GFAP to indicate the interfaces of the grafts and the native tissues because tissue-resident astrocytes contributed to glial scar formation in injury [46, 47] (Figure 2(j)).

Next, to examine whether the collagen microgels themselves could assist neural tissue regrowth, acellular col-DNA microbeads were synthesized and implanted into the transected spinal cord cavities (Figure 2(l)). Eight weeks later, the grafted collagen beads formed a translucent chunk, indicating the lack of cells (Figure 2(m)). Limited axons were found in the grafts while more abundant axons were located at the interfaces (along with the overlapped frames 1 and 3 and frames 2 and 6) or in the native tissues, which were revealed by the GFAP distribution (Figures 2(n)–2(p)).

Further, we postulated that the collagen gel as beads provide spaces that allow cell infiltration and migration, thus promoting neural regeneration and angiogenesis. To test the hypothesis, we synthesized the bulk collagen gel by incubating the mixed components of collagen (2 mg/mL) and ssDNA (0.2 mg/mL) in a columnar mold matching the size of the transected spinal cord (Figures 2(e) and 2(f)). Both the cell-laden and acellular collagen gels were transplanted, and their regeneration capacities were investigated by H&E staining and immunostaining of NF and GFAP (Figures 2(q)–2(u) for cell-laden gel and Figures 2(v)–2(z) for acellular gel implantation).

Among the four transplantation groups using collagen as the major scaffold, the grafted tissues displayed differential courses of NF+ axon development. The cell-laden collagen bead group gained the highest level of axon growth and the bead grafts over the bulk groups in both cell-laden and acellular comparisons. Furthermore, the pan-neuron marker, TUJ1, was observed in the grafted tissues together with the native tissues. Abundant thin filament strings enclosed with stretched nuclei were observed in the cell-laden collagen bead graft, but barely in other grafts (Figures S6). The results suggest that transplantation of bead-encapsulated NPCs contributes to neural tissue growth after injury. In terms of material configuration, microbead assembly increases the regeneration ability when compared with gel chunks.

Additionally, the sectioned slices were stained with the anti-CD31 antibody to mark the blood vessels which provide a nutritionally favorable environment for neurogenesis [48–50]. The signals of CD31 were found at the edges of some cavity-like structures within the grafts (Figure S8). However, for both the cell-laden collagen bead transplant and the acellular bulk collagen transplant, no obvious differences were observed in the CD31+ morphology and quantity. This may suggest that angiogenesis is not the key factor in these cases to promote neural regeneration.

### 2.2. Injection of Cell-Laden Matrigel Beads Induced Robust Axonal Regeneration

**Figure 3:**
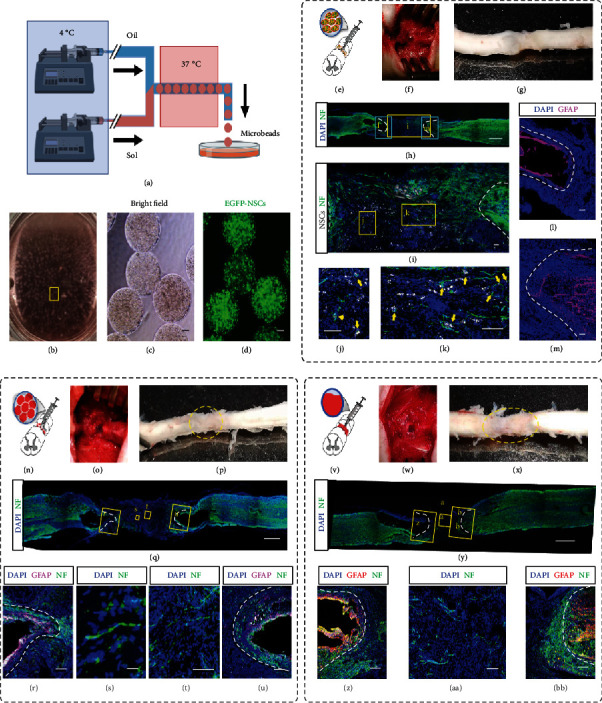
Implantation of cell-laden and acellular Matrigel beads to intertransects of spinal cords. (a) Schematic of the production of cell-laden Matrigel beads by using the CTM technique. (b) Collected Matrigel beads in DMEM/F12 medium. The oil residue was removed by gentle pipetting. (c, d) Bright-field (c) and fluorescence (d) images of the collected monodisperse cell-laden Matrigel beads (~500 *μ*m in diameter). Each bead was encapsulated with EGFP-labeled NPCs. (e) Schematic of injection of cell-laden Matrigel beads in (d) to the intertransects of a spinal cord. (f) The cell-laden Matrigel beads were injected into the space where a 4-5 mm spinal cord tissue in length had been cut and removed. (g) The harvested spinal cord tissue at 8 weeks postimplantation. The dotted circle marks the boundary of implanted beads. (h–m) Confocal sagittal imaging of the immunostained spinal cord tissue. DAPI shows the nuclei. Neurofilament (NF, green, labeled by anti-NF antibody) indicates the axons (h–k). The enlarged area in (h) shows the EGFP-labeled NPCs (pseudocolor in grayscale) and axons (green) (i). Zoom-in view shows the nonoverlapped but physically proximate EGFP-NPCs (indicated by yellow arrows) and axons (j, k). White dot lines in (i) indicate the interfaces between the grafted and host tissue. (l, m) The fluorescence distribution of GFAP, the marker for astrocytes, and DAPI. White dot lines represent the boundaries of the glial scar. (n–u) Schematic (n) and follow-up characterization (o–u) of injection of acellular Matrigel beads to the intertransects of a spinal cord. (v–BB) Schematic (v) and follow-up characterization (w–BB) of implantation of molded acellular Matrigel trunk to the intertransects of a spinal cord. Scale bars, 20 *μ*m (s, t), 100 *μ*m (c, d, i, j, k, l, m, r, u, z, AA, BB), and 1 mm (h, q, y).

Matrigel is rich in various proteins that exist in the extracellular matrix of the CNS [51–53]. Therefore, we adopted Matrigel as a substitution for collagen to produce the microbeads by the CTM technique (Figures 3(a)–3(d)). We labeled the ReNcells, a commercial NPC line, with EGFP, which were subsequently for producing the cell-laden Matrigel beads (Figures 3(c) and 3(d)). Eight weeks later, the immunofluorescent examination (Figures 3(e)–3(m)) showed that the emerged axons bridged the transected spinal cord and exhibited a sharp and abundant fibrous morphology (Figures 3(i)–3(k)). However, the grafted NPCs did not participate in the neural regeneration directly, though they were found closely located with the axons (Figures 3(j) and 3(k), yellow arrows). This is also corresponding to the consensus that transplanted stem cells facilitate tissue regeneration by the paracrine effect [54].

When acellular Matrigel beads were injected, only thin axonal fibers were observed that were less abundant and robust than those in the cell-laden bead graft (Figures 3(n)–3(u)). Moreover, a Matrigel chunk was solidified in a mold that matched the size of the transected cavity for implantation. However, the gelled Matrigel was friable and easily fractured when being transferred. As an alternate solution, liquid Matrigel was injected into the cavity and gelled in situ. The immunostaining was performed after 8 weeks (Figures 3(v)–3(bb)). A few axons were observed inside the injected gel, but the functional connection was barely seen (Figures 3(y) and 3(aa)). Of note is that the gap between the rostral and caudal interfaces of transection was always shortened in the liquid Matrigel injection group. We postulate that the solidified microbeads served as stuffing to keep the fresh transects in position, whereas the liquid Matrigel failed to function likewise before gelation, which needed 20-30 min. The axon regeneration was less as significant and robust as in the cell-laden Matrigel beads.

### 2.3. Implantation of Cerebral Organoid Promoted Axon Regeneration and Retained Exogenous Neural Networks

The former sections demonstrated the regenerative capacities of freshly engineered extracellular materials in various configurations. Cell-laden microbeads proved supreme performance in promoting neural regeneration compared with their counterparts. However, it remains unaddressed whether the microtissues as therapeutic agents should be prematured or preorganized *in vitro* before transplantation [55–57]. Herein, organoids, as the most advanced representative of *in vitro* partially matured microtissues, were investigated for their role in regeneration.

**Figure 4:**
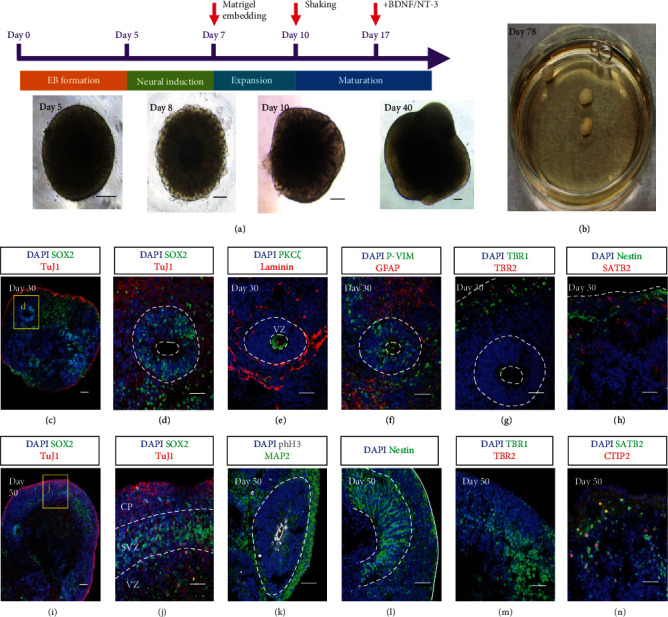
Generation of cerebral organoids. (a) Schematic diagrams illustrating the protocol to generate cerebral organoids. Differential interface contrast (DIC) images in the lower panel show the morphology of the organoid changes on day 5, day 8, day 10, and day 40. (b) The photo displays three cerebral organoids at day 78 in a 3 cm dish. (c–h) Confocal images of the cryosections of cerebral organoids at day 30. The organoids express the neural stem cell marker SOX2 in a rosette structure and pan neural marker TuJ1 in the region beyond the rosette (c, d; (d) is the magnified image of the boxed area in (c)). The apical- (PKC*ξ*) basal (Laminin) structure indicates polarized cortical neuroepithelia (e). Radial glial cells expressing the combination of P-Vimentin (P-VIM) and GFAP distribute both in and out of the proliferative zone (f). Deep-layer neuron marker TBR1 and intermediate progenitor cell marker TBR2 are found at the edge of the organoids (g). Late-born neurons are marked by SATB2, while neural stem cells are revealed by Nestin within the same region (h). (i–n) Confocal images of the cryosections of cerebral organoids at day 50. A lobe structure with an identified SOX2-expressing layer is characterized by the cortical plate (CP), subventricular zone (SVZ), and ventricular zone (VZ) (i–j). Apical mitoses are revealed by phospho-histone H3 in a polarized rosette surrounded by MAP2+ cells (k). The Nestin+ cells are abundant both in the rosette and along the lobe periphery (l). Neurons are expressing TBR1, but TBR2 is found to be scarce (m). The signals of the surface cortical neuron marker SATB2 and deep-layer cortical neuron marker CTIP2 are shown (n). Scale bars, 50 *μ*m (c, d, e, f, g, h, i, j, k, l, m, n) and 200 *μ*m (a).

Both the cerebral cortex and spinal cord tissues belong to the CNS and share similarities in physiology and mechanical properties [58]. Therefore, matured cerebral organoids were employed to repair spinal cord transects. COs were generated by following an established protocol [31]. Human embryonic stem cells (hESCs) were expanded, harvested, and then reseeded in ultralow attachment wells to generate embryonic bodies (EBs). After the neural induction process, the neural spheroids were embedded into Matrigel and then cultured for expansion and maturation to generate cerebral organoids (Figures 4(a) and 4(b)). Immunostaining of cryosectioned tissue slices was performed to validate the identity of produced cerebral organoids at day 30. The proliferative zone was marked by SOX2-expressing cells (Figure 4(d)), and the typical apical-basal structure was identified by the polarized neural rosette with the apical protein PKC*ξ* and the Laminin+ basal surface (Figure 4(e)). One of the radial glial cell markers, phosphorylated Vimentin, was found near the apical of the rosettes and in their extended regions, which were surrounded by astrocytes (GFAP) (Figure 4(f)). The deep-layer neuron marker TBR1 and intermediate progenitor cell marker TBR2 were found at the edge of the organoids (Figure 4(g)), together with the surface cortical neuron marker SATB2 and the neural stem cell marker Nestin (Figure 4(h)). By day 50, the neuroepithelial lobe had formed and the different layers, the cortical plate (CP), subventricular zone (SVZ), and ventricular zone (VZ), were distinguished by the distributions of SOX2- and TuJ1-expressing cells (Figures 4(i) and 4(j)). Some rosettes remained. The cells expressing proliferation marker phospho-histone H3 (phH3) were located near the apical direction while other cells expressing the neuronal marker MAP2 were located in the surrounding areas of the rosettes (Figure 4(k)). Nestin-positive neural stem cells were preserved (Figure 4(l)). TBR1 was significantly expressed, but the TBR2 was barely to be found (Figure 4(m)). Additionally, the SATB2 and deep-layer cortical neuron marker CTIP2 were stained within the organoids (Figure 4(n)), indicating the forebrain identities of the COs.

**Figure 5:**
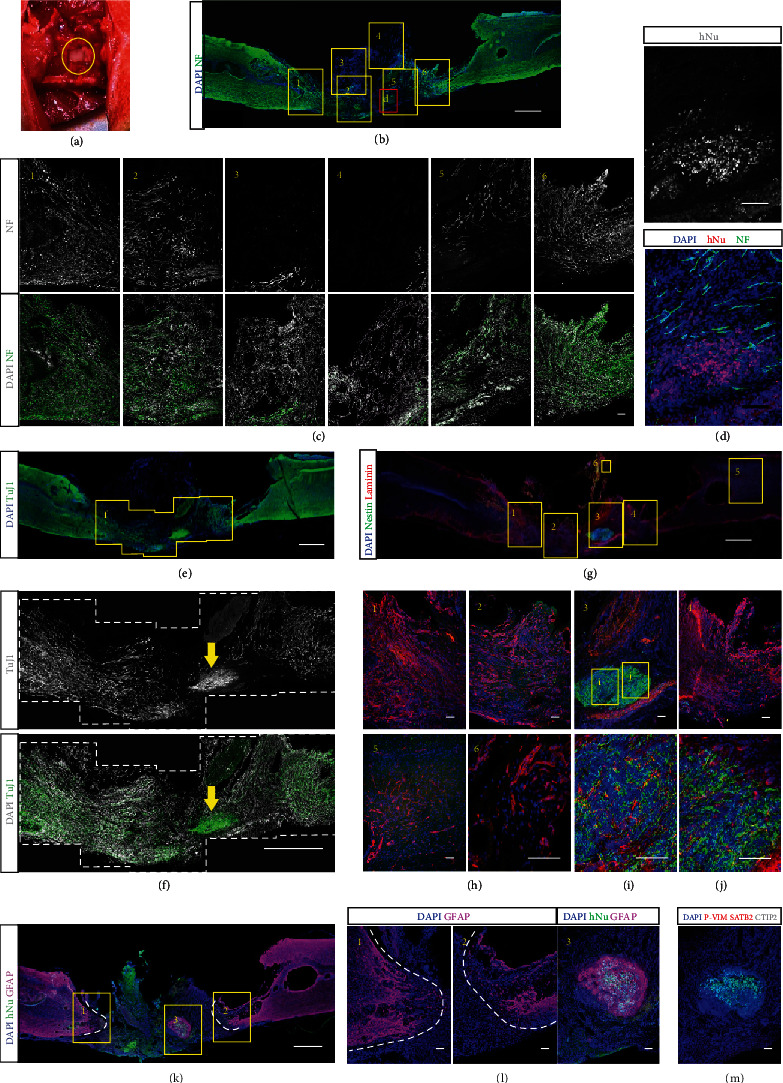
Transplantation of human iPSC-derived cerebral organoids into the transected spinal cord. (a) A large cerebral organoid (~5 mm in diameter) was inserted into the space where 5 mm long spinal cord tissue had been cut and removed. (b–d) Confocal sagittal images of the cryosection of a spinal cord tissue 8 weeks after receiving the organoid transplantation. Images 1, 2, 3, 4, 5, and 6 of (c) are the enlarged views in (b), showing the distribution of axons (NF, green) and nuclei (DAPI). The boxed area (d) in red is magnified, which shows the aggregation of human cells marked by human nuclear antigen (hNu) (d). (e, f) Confocal sagittal images of the immunostaining of the pan-neural marker TuJ1. The enclosed area in (e) is enlarged in (f). Yellow arrows indicate the position of the human cell aggregate. (g–j) Immunostaining images of Laminin, indicative of possible angiogenesis, and Nestin, indicative of neural stem cells, of the spinal cord tissue with an implanted cerebral organoid. The images 1-6 in (h) are the enlarged areas in (g). The boxed areas in 3 in (h) are magnified in (i) and (j) to show the detailed distribution of Laminin and Nestin. (k, l) Confocal images show the distribution of hNu, GFAP, and SATB2, indicative of human cells, astrocytes, and surface cortical neurons, respectively. The enlarged areas in (k) are shown in (l) and (m). The distributions of GFAP+ cells (astrocytes) are shown in 1 and 2 in (l), while the human cell cluster labeled by hNu is shown in 3 in (l). The significant signals of SATB2 are observed within the human cell cluster, but those of P-VIM and CTIP2 are found to be scarce (m). Scale bars, 100 *μ*m (c, d, h, i, j, l, m) and 1 mm (b, e, f, k).

Next, the transplantation of COs was explored to promote tissue regeneration and neuronal connection (Figure 5(a)). 8 weeks after transplantation, the spinal cord tissues were harvested and the sectioned slices were incubated with the axonal marker NF (Figure 5(b)). The CO fused with the recipient spinal cord tissue, but the dorsal part of the organoid degenerated significantly, generating cavities there (Figure 5(c), frames 3 and 4). The axons robustly extended into the grafted organoid from both rostral and caudal directions (Figures 5(b) and 5(c)). A cluster of cells was identified as human-derived cells by immunostaining with the human nuclear antigen-antibody (hNu) (Figure 5(d)). The human cell cluster highly expressed the pan-neuron maker TuJ1 (Figures 5(e) and 5(f), yellow arrow) and neural stem cell marker Nestin (Figures 5(g)–5(j)), suggesting the immature identity of these cells. In addition, astrogenesis was observed along with the human cell cluster, when the interfaces of the graft and host tissues were labeled with GFAP, the marker for astrocytes (Figures 5(k) and 5(l)). SATB2 was observed abundant within the human cell cluster, but no CTIP2 was found (Figure 5(m)), implying that only specific types of neurons could survive in the environment of the spinal cord. It is worthy to notice that the Laminin deposit, the marker of blood vessels, was significant within the grafted cerebral organoid and in the human cell cluster as well (Figures 5(g) and 5(h)).

### 2.4. Immune Responses Triggered among the Transplantations

**Figure 6:**
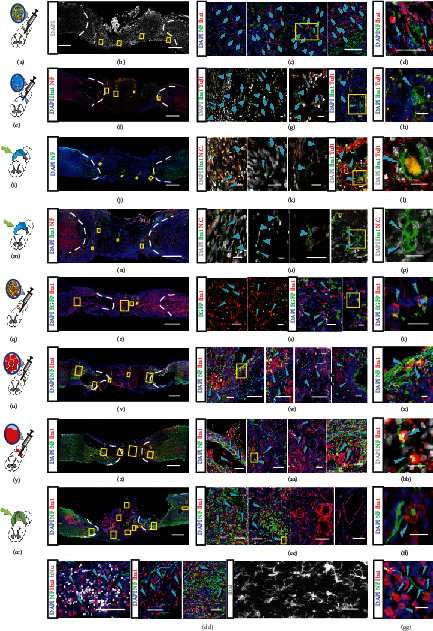
The immune responses of the spinal cord tissues after receiving transplantation of different types of materials. (a–d) Confocal images show the Iba1+ microglial cells/macrophages in the spinal cord tissue and transect receiving cell-laden col-DNA bead transplantation: schematic of bead transplantation (a), the large-scale image of DAPI (b), and the magnification and immunostaining of inactivated Iba1+ cells (cyan arrows) in boxes 1-4. The activated iba1+ cells are marked with cyan lightning bolts and enlarged in (d). (e–h) Confocal images of the Iba1+ cells in the col-DNA bead transplantation group. Schematic shows the transplantation (e) and the immunostaining of Iba1 and NF (f). Enlarged areas marked with 1, 2, 3, and 4, highlighting that the Iba1+ cells (cyan arrows) and the activated one (cyan lightning bolts) are shown in 1, 2, 3, and 4 in (g) and (h). (i–l) Confocal images show the immune cells and neurons in the cell-laden collagen bulk gel implantation group. Schematic illustrates the implantation (i). The positions of the enlarged areas in (k), showing the Iba1+ cells (cyan arrows) and the activated Iba1+ cells (cyan lightning bolts), are indicated in the large-scale image of the DAPI and NF immunostaining (j). The activated Iba1 cells engulfing cell debris are magnified as shown in (l). N.C. represents the negative control. (m–p) Confocal images show the Iba1+ cells, NF+ neurons, and TuJ1+ neurons in the acellular collagen bulk gel implantation group. Schematic illustrates the implantation (m), and the areas in (n) are magnified in (o). The Iba1+ cells are marked with cyan arrows, and the activated Iba1+ cells zoomed-in (p) are marked with cyan lightning bolts. N.C. represents the negative control. (q–t) Immunostaining images show Iba1 and EGFP signals in the spinal cord tissue transplanted with the cell-laden Matrigel beads. Schematic shows the operation of cell-laden Matrigel bead injection (q). The large-scale image shows the spinal cord tissue injected with cell-laden Matrigel beads and stained with Iba1 and EGFP (r). The enlarged images show the framed areas in the rostral region (1 in (s)) and inside the graft (2-4 in (s)). The transplanted NSCs were labeled with EGFP, and the immune cells were stained for Iba1. Higher magnification images show some inactivated Iba1+ cells (s; cyan arrows). The transplanted EGFP-labeled cells (s, t; green arrows) are physically proximate to the activated Iba1+ cells (s; 3-4; t, cyan lightning bolts). (u–x) Immunostaining images of the neurons (NF) and microglial cells/macrophages (Iba1) of the spinal cord tissue after an acellular Matrigel bead injection. Schematic illustrates the injection of Matrigel beads into the transected spinal cord (u). Magnified areas in (v) are shown in 1-5 in (w). The Iba1+ cells are labeled with cyan arrows, while the activated Iba1+ cells engulfing neuronal debris are indicated by cyan lightning bolts (x). (y–BB) Confocal images show the immunostaining of neurons (NF) and immune cells (Iba1) in the transected spinal cord receiving liquified Matrigel injection. Schematic indicates the operation (y). Immunostaining image shows the injection site and the rostral and caudal regions (z). The boxed areas in (z) are shown in 1, 2, 3, and 4 in (AA). Regular Iba1+ cells are indicated by cyan arrows, while the activated Iba1+ cells are labeled by cyan lightning bolts. Two of the activated Iba1+ cells in the boxed area in 2 in (AA) are shown in the magnified image of (BB). (CC–GG) Confocal images of the neuron (NF) and the microglial cells/macrophages (Iba1) of the spinal cord with cerebral organoid insertion. Schematic illustrates the implantation of a cerebral organoid into the transected spinal cord (CC). The immunostaining shows the distribution of NF and Iba1 within the implanted cerebral organoid and its surroundings (DD). The enlarged areas in (DD) are shown in 1-8 in (EE), (FF), and (GG). The Iba1+ cells in the host tissue that do not exhibit activated morphology are enlarged and shown in 8 in (EE) that is distant from the graft. Regular Iba1+ cells are indicated by cyan arrows. Some of the activated Iba1+ cells are shown in (GG). Another activated Iba1+ cell engulfing the neuronal debris is shown in (FF), which is the enlarged area of 2 in (EE). All the activated Iba1+ cells are labeled by cyan lightning bolts. Scale bars, 20 *μ*m (d, g, h, k, l, o, p, s, t, z, BB, FF, GG), 100 *μ*m (c, w, AA, EE), and 1 mm (f, j, n, r, v, z, DD).

Immune response aroused by hemorrhage after acute damage of spinal cord tissue and materials or cells transplanted is considered detrimental and destructive to tissue regeneration [7, 59, 60]. The microglia/macrophage reactions are subject to immune activity in spinal cord injury [61]. To evaluate the immune response induced by the damage and the exogenous materials, we marked the microglia/macrophages by revealing the Iba1 existence. The Iba1+ cells were found in the graft formed by 5the transplanted cell-laden collagen beads, most of which had the inactivated morphology (Figures 6(a)–6(d), cyan arrows). Some of the activated Iba1+ cells colocalized with axons (NF) within the grafts (Figure 6(d), cyan lightning bolts to mark the activated Iba1 cells). A similar situation was observed in the transplantation of the acellular collagen beads (Figures 6(e)–6(h)). The number of the Iba1+ cells reduced significantly in both groups that received cell-laden bulk collagen and acellular bulk collagen implantations (Figures 6(i)–6(p)). In these two groups, Iba1+ cells exhibiting reactive morphology were found at the boundary region of the graft (Figures 6(l) and 6(p), cyan lightning bolts), some of which were engulfing the neuronal debris conjugated with the TuJ1 antibody (Figure 6(l), cyan lightning bolt). It suggested that though some immune activities were induced at the interfaces, the collagen-based grafts triggered the activation of Iba1+ cells but remained at a low level, indicating a beneficial environment for tissue repair with generated low immune response.

In the cell-laden Matrigel beads grafts, the number of the immune cells was increased by more than 3-fold when compared with the collagen counterpart (Figures 6(q)–6(t)). Moreover, the Iba1+ cells were physically proximate to the EGFP-labeled human NPCs (Figures 6(s) and 6(t); green arrows mark the NPCs, and cyan lightning bolts label the activated Iba1 cells), suggesting being attacked by the immune cells. Equivalent amounts of Iba1+ cells were observed in the acellular Matrigel beads grafts (Figures 6(u)–6(x)) and the liquid Matrigel (Figures 6(y)–6(bb)) injections. Among these groups, Iba1+ cells infiltrated into the grafts activated and accumulated at the interfaces where some large cavities emerged. These activated immune cells may have resulted in cavities by clearing the axon and other cell debris (Figures 6(w), 6(x), 6(aa), and 6(bb), cyan lightning bolts).

In the CO transplantation, though the axons were robustly generated, the Iba1+ cells were high in number and the majority of them were activated according to the morphological hallmark—a plump soma with a few thick protrusions [62] (Figures 6(cc)–6(gg), cyan lightning bolts). On the dorsal side of the graft, there were Iba1+ cells along with the inner layers of the cavities (Figure 6(ee), frames 3 and 4). Notably, albeit the immune cells infiltrated into the human cell cluster, they did not accumulate in or attack the engrafted cells (Figure 6(ee), frame 5). The exclusive ramified morphology of the Iba1+ cells in the region far from the graft remained in an inactivated state (Figure 6(ee), frame 8).

### 2.5. Quantifications of the Functional Recovery, Neuronal Regeneration, and Immune Response

**Figure 7:**
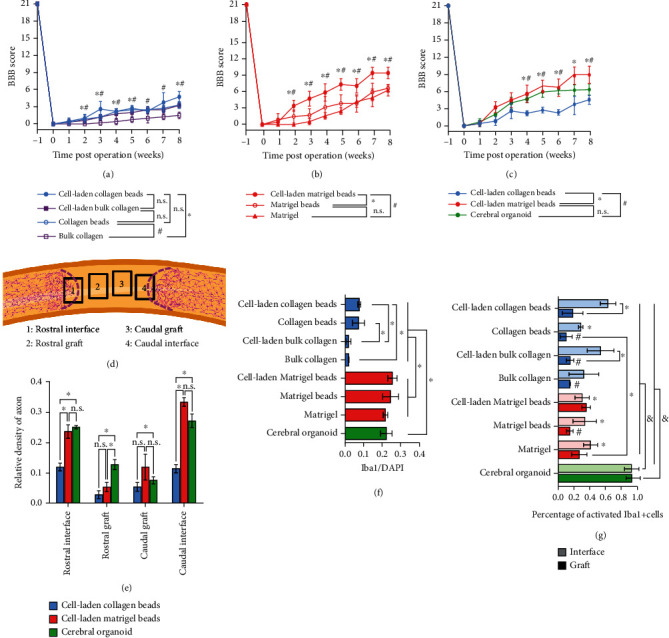
Quantification of the locomotor function recovery, neuronal regeneration, and abundance of immune cells. (a) The locomotor behavior of the collagen-based materials in the form of col-DNA beads and bulk gel, either cell-laden or acellular, is accessed via Basso, Beattie, and Bresnahan (BBB) locomotor scale method. Unpaired two-tailed Student's *t*-test was applied (*n* ≥ 5/group). (b) The locomotor function recovery of the transplantation of the Matrigel-based materials in the form of cell-laden beads, acellular beads, and liquid Matrigel. Unpaired two-tailed Student's *t*-test was applied (*n* ≥ 5/group). (c) The BBB locomotor scores are compared within the groups transplanted with cell-laden collagen beads, cell-laden Matrigel beads, and a cerebral organoid. Unpaired two-tailed Student's *t*-test was applied (*n* ≥ 3/group). (d) Schematic indicates the areas used to quantify neuronal regeneration at the rostral interface of the graft and the host tissues (1), the rostral part of the graft (2), the caudal part of the graft (3), and the caudal interface of the graft and the host tissues (4). The magenta dot lines indicate the boundaries of the GFAP+ cells from the host tissue, showing the interfaces between the host tissue and grafts. (e) Quantification of the signal density of axons within the four areas among the cell-laden collagen beads, cell-laden Matrigel beads, and cerebral organoid transplantations. Paired two-tailed Student's *t*-test was applied (*n* = 3/group). (f) Quantification of the Iba1+ cells within the grafts. Paired two-tailed Student's *t*-test was applied (*n* = 3/group). (g) Quantification of the activated Iba1+ cells among all the Iba1 cells within the regions of interfaces and grafts. ∗ above the columns represents the significant differences when comparing the “interface region” with the group of “cell-laden collagen beads”, while other ∗ represents the significant differences between the indicated columns. # above the columns represents the significant differences when comparing with the “grafts” region of the group of “cell-laden Matrigel beads”. & above the columns of the group of “cerebral organoid” indicates the significant differences when compared with all other columns. ∗, #, and & represent the *p* value < 0.05 in (a, b, c, e, f, g), and the n.s. represents no statistically significant difference. Data are represented as means ± SDs.

The BBB score is a widely used method to evaluate the functional recovery of the hindlimbs in the rats which received spinal cord injury. For the collagen-based implantation groups, though the scores of the cell-laden beads group (4.75 ± 0.83) were the highest from 4-8 weeks postimplantation, there were no statistically significant differences when compared with the acellular beads (3.33 ± 0.47) or the cell-laden bulk collagen groups (3.25 ± 0.43), except for the acellular bulk gel group (1.50 ± 0.50) (Figure 7(a)). When Matrigel was applied as the implanted materials, the cell-laden beads achieved the best functional recovery (9.00 ± 0.82, week 8). The rats were capable to do plantar weight support and steps occasionally. This is corresponding to the immunostaining of axons which largely existed in the grafts and bridged the transected area (Figures 3(h) and 3(i)). Comparatively, the quantified results show compromised functional recovery from weeks 2-8 (Figure 7(b)) in the rats that received acellular bead (6.33 ± 0.47, week 8) or liquid Matrigel (6.00 ± 0.82, week 8) transplantations. The BBB scores of the CO implantation group (7.00 ± 0.82) were compared with the scores from the cell-laden collagen beads and the cell-laden Matrigel beads. No significant difference was found between the CO group and the cell-laden Matrigel bead group, but both were significantly improved than the cell-laden collagen bead group (Figure 7(c)). The comparison indicated that there was a deficiency in the neuronal connections when applying the CO transplantation, as revealed by the axonal immunostaining that the neuronal fibers did not fully connect and the engrafted organized cells did not contribute to the neuronal regeneration (Figures 5(b) and 5(c)).

The density of the axon signals was from four areas: two near the rostral and caudal interfaces revealed by the astrocyte accumulation (GFAP) (Figure 7(d), frames 1 and 4) and the other two within the grafts (Figure 7(d), frames 2 and 3). The results showed that the axons significantly appeared in the grafts at the interfaces in the cell-laden Matrigel bead group and CO group, between which there were no significant differences (Figure 7(e)). However, in the grafts, the relative density of axons in all three groups was lower than that in the regions of rostral (1) and caudal (4) interfaces, though the transplantation of CO achieved obvious localized neuronal regeneration (Figure 7(e)).

Finally, the number of Iba1-expressing microglial cells/macrophages inside the grafts was quantified to evaluate the immune response during tissue regeneration. The cell number was normalized to DAPI counts. The Iba1+ cells in the transplanted collagen bead groups (regardless of containing cells or not) were more than those in the bulk collagen groups (Figure 7(f)), suggesting that the beads provided spaces for infiltration or migration of the immune cells. The number of the Iba1-expressing cells greatly increased in the Matrigel-based implants, but no statistically significant differences exist (Figure 7(f)). In addition, in the CO groups, the Iba1+ cells were in a large number, resembling the Matrigel-based groups (Figure 7(f)). The number of Iba1+ cells was supposed to describe the immune response that impeded neural regeneration. However, a significant amount of Iba1+ cells were found in the CO implantations. Meanwhile, robust axonal regeneration and locomotor function recovery were observed in both cell-laden Matrigel bead and CO implantations. The activated Iba1+ cells were used to estimate the immune response according to the amoeboid morphology rather than the ramified one [62], which was found high in number at the interface of the grafts and the host tissues in all groups. The collagen grafts contained the fewest activated Iba1+ cells (Figure 7(g)). The activated Iba1+ cells were largely observed in the grafts of cell-laden Matrigel beads, which showed optimum axonal regeneration and functional recovery, compared with the grafts of collagen beads, cell-laden bulk collagen, bulk collagen, and acellular Matrigel beads (Figure 7(g)). Interestingly, the amoeboid Iba1+ cells existed in great amounts and spread over the transplanted CO, which was not capable to develop Iba1+ cells during *in vitro* culture [31, 63, 64] (Figure 7(g)). These observations validate the positive effect of the reactive immune cells during neural tissue regeneration and meanwhile imply the optimized neural regeneration by employing CNS organoids as the agent.

## 3. Discussion

The optimal materials and their configuration in spinal cord regeneration are yet to be determined. When and how to transplant therapeutic stem cells are continuously under debate in the community. This study was aimed at addressing whether transplanting cell aggregates with organized cytoarchitecture, i.e., organoids, would improve the regeneration outcome. The performance was compared of two different materials, collagen and Matrigel; two configurations, beads assembly and bulk gel; and two ways of cell infiltration, exogenous cell implantation and endogenous cell migration. We will discuss the findings from the following viewpoints:

### 3.1. Exogenous Cells Promote the Neuronal Regeneration

It was found that the axonal regeneration and hindlimb locomotor function recovery in the rats that received the *in vitro* organized CO transplantation were comparable to the results obtained from the cell-laden Matrigel bead implantation, both better than the acellular implantation. The results suggested the positive functions of the grafted human cells. Subsequently, the cells transfected with the EGFP allowed us to observe that they did not directly give rise to neurons. Given that many axons in the grafts colocalized with the human cells (Figures 2(j) and 2(k)), the paracrine signals emerging from the exogenous cells might have induced the sprouts of host neurons extending into the grafts.

### 3.2. The Configuration and Materials of the Grafts Affect Neural Tissue Regeneration

Our previous study shows that the collagen microgels worked better than the bulk gel in wound repair and liver tissue regeneration [44]. In the transplanted cell-laden bulk collagen, the regenerated axons formed a bundle but did not form a connection along the rostral-caudal direction. In the transplanted collagen bead assembly, the axons were found more evenly spread in parallel with the cord axis (Figure 1(k)).

To investigate the effects of encapsulated cells, a wealth of the signals of NF and TuJ1 were observed in the engrafted beads but not in the acellular collagen bulk. It demonstrated that the interspaces brought by the acellular beads facilitated the migration of host cells. A similar scenario was observed in the immunostaining of the Iba1 expressing cells, of which the numbers were significantly higher in the transplanted beads regardless of carrying cells or not. However, the usage of the beads also raised the problem that the spaces they created were not linearly arranged. This might have resulted in the lack of continuous NF fibers in the engrafted cell-laden beads.

### 3.3. Organoids as *In Vitro* Matured Cell Organization Facilitate Regeneration of Spinal Cord Tissue

It was intriguing to find that apart from the degenerated dorsal part, the implanted cerebral organoids fused with the recipient tissue on the ventral side, and a cluster reminiscent of the exogenous human cells merged into the regenerated rat spinal cord tissue. A mixture of the human cells remained as neural stem cells (Nestin), astrocytes (GFAP), and the superficial cortical neurons (SATB2), but not the proliferating radial glial cells (P-VIM) or the deep-layer cortical neurons (CTIP2). It was reported that the successful fusion of the neural tissues is selective. For instance, the retinofugal neurons from the human retinal organoids had a higher affinity to the lateral geniculate nucleus than the olfactory bulb isolated from the mouse brain [65]. The cortical organoids tended to fuse with the spinal cord organoids rather than the skeleton muscle organoids [66]. These reports imply that the local environment supports the survival of specific types of neurons. In future studies, spinal cord organoids should be transplanted necessarily to achieve better spinal cord tissue regeneration.

The CO served as a bulk of organized cell aggregate in this study. The bulk organoid stopped bleeding in the transects, which had benefited the repair process. Further, microglia in the activated state reached the highest density in the organoid transplantation group. Based on the positive effect of activated microglia in promoting the growth of organoid-derived neural tissue in injury [67–70], the high density may reflect increased regeneration activity in the organoid group. In the immune environment study, the organoid transplant received the highest Iba1 enrichment and activation. It provided evidence to support using neural organoids, or preorganized cell populations, as the implanted agents. However, inspired by the increased performance from bead configurations, smaller and multiplex organoid assembly rather than single, bulky organoids might be employed to further improve the axon regeneration in spinal cord repair.

### 3.4. Neural Regeneration Was Not Significantly Affected by the Immune Response Which May Be Triggered by Matrigel

Matrigel is a mixture of various proteins derived from Engelbreth-Holm-Swarm (EHS) mouse sarcoma. It contains many unidentified molecules related to tumor development and growth that could be potential antigens. The generation of COs needed to be embedded in Matrigel. An increase in the number of Iba1-positive cells in the Matrigel-based transplantation group and the CO transplantation group was observed, suggesting that the immune response was induced by Matrigel.

Although the implanted cells within Matrigel beads were found to be closely related to Iba1+ cells, the axon regrowth was not affected by the immune cells. The exogenous cells did not differentiate into neurons and directly participate in the reestablishment of neuronal connections. Meanwhile, the human cells from the implanted CO were neglected by the Iba1+ cells which were distributed evenly in the implants. This evidence demonstrated that the immune response caused by exogenous human cells was not comparable to that triggered by Matrigel. Therefore, there is a great need of developing a chemical-defined substitution for Matrigel in future clinical use.

It is still early to conclude the advantages of organoids, as the representative configuration of premature cell-laden structures, against fresh, unmatured cell-laden microgels in SCI repair. More experimental validations are required with high controllability on variables, including the cell counts, the scaffolding materials, the size and shape of the cell-laden configurations, and prolonged observation posttransplantation. Particularly, to further elucidate the regulatory cues on CNS organoids toward improved SCI repair, submillimeter-sized CNS organoids of different developmental stages should be studied in cavity-assembly manners. The bead assembly is suggested to improve the transplantation volume homogeneity and host cell infiltration by providing the interstitial spaces among beads (i.e., small organoids). The manner of cell organization during CNS organoid growth could be manipulated by incorporating microfilament as the supporting scaffold which guided the formation of rod-like organoids [71], providing the feasibility of producing injectable microneural tissue with a linear arrangement of neural cells that resemble spinal cord structure. Though organoids augment microglial participation in neural regeneration, the level of *in vitro* cell complex maturation for optimized host-guest integration needs experimental validation. Further studies will elaborate on the operations and choice of therapeutic agents for SCI repair.

## 4. Materials and Methods

### 4.1. Human Cell Culture and Viral Labeling

The human iPSC line was purchased from the Cell Inspire Biotechnology Co., Ltd. in Shenzhen, China, and maintained in mTeSR1™ completed medium (STEMCELL Technologies). The human ESC line H1 was purchased from the MeisenCTCC in Zhejiang, China, and maintained in mTeSR™ Plus completed medium (STEMCELL Technologies). The iPSC and ESC lines were cultured in 6-well plates coated with the Matrigel (Corning) diluted with 1% in DMEM/F12 (Gibco). Human NPC line ReNcell® VM (Sigma-Aldrich) was maintained in the ReNcell® NSC maintenance media (Sigma-Aldrich) supplied with 20 ng/mL basic fibroblast growth factor (bFGF) (CHEMICON) and 20 ng/mL epidermal growth factor (EGF) (CHEMICON). For NPC generation, we applied the STEMdiff™ SMADi Neural Induction Kit (STEMCELL Technologies) to induce the iPSCs to differentiate into NPCs following the manufactory protocol. The neural progenitors were maintained in the STEMdiff™ Neural Progenitor Medium (STEMCELL Technologies). The lentivirus solution (Hanbio) was incubated with the ReNcell to label the cells with EGFP. The cells that failed to transfect were eliminated by the treatment of puromycin (MCE). All the cells were maintained at 37°C in an incubator with a 5% CO_2_ supply.

### 4.2. Production of Microbeads and Bulk Gels

The cell-laden col-DNA collagen beads were produced as previously described [44]. Briefly, the ultralong ssDNA was synthesized by the rolling circle amplification (RCA) method. 10 *μ*L of the RCA product or complementary ssDNA (c-RCA) (2 mg/mL for stock solution) was mixed with 100 *μ*L of collagen type I (2 mg/mL) and incubated at 4°C for 30 minutes. The final concentration of DNA was 0.2 mg/mL. The pH of the mixture was adjusted to 7.0 to obtain the pregelled RCA-col solution. The induced NPCs were dissociated by incubating with Accutase (STEMCELL Technologies) and centrifuged to discard the supernatant. The cell pellet of 1 × 10^6^ cells was mixed with the pregelled RCA-col solution, which was loaded into a syringe connected to a three-way polydimethylsiloxane (PDMS) made T junction with polytetrafluoroethylene (PTFE) tubing. The fluorocarbon oil HEF7100 (3 M) was aspirated into another syringe and connected to the T junction. The injection flow rates were 0.3 mL/min for oil and 0.2 mL/min for the pregelled RCA-col solution. The beads had a diameter of 800 *μ*m and contained approximately 1090 cells in each bead. This protocol was applied to produce acellular collagen beads. For the production of cell-laden Matrigel beads, the cell pellet of 3 × 10^6^ EGFP-labeled ReNcells was mixed with ~100 *μ*L of Matrigel and loaded into a syringe which was immediately placed into a 4°C refrigerator to avoid gelation. The HEF7100 was used to separate the Matrigel solution in the microfluidic tubing at the flow rates of 0.3 mL/min for oil and 0.2 mL/min for Matrigel (containing cells or not). The Matrigel beads had a diameter of 600 *μ*m and contained approximately 1961 cells in each bead. For the generation of cell-laden bulk collagen gel, the cell pellet of 1 × 10^5^ cells was mixed with the pregelled RCA-col solution and incubated at 37°C for at least 30 minutes in a columnar mold. The same method was also applied for generating acellular bulk collagen gel.

### 4.3. Generation of Cerebral Organoids

The cerebral organoids were generated by the STEMdiff™ Cerebral Organoid Kit (STEMCELL Technologies) following the manufactory protocol. Briefly, at day 0, the H1 colonies were detached from the Matrigel-coated culture surface by incubating with the Gentle Cell Dissociation Reagent (STEMCELL Technologies) and then resuspended in the EB seeding Medium (EB Formation Medium containing 10 *μ*M of Y27632 (MCE)). H1 cell suspension was added into the 96-well ultralow attachment V-bottom plate (Sumitomo) with 9000 cells for each well to generate EB. On day 5, the EB formation medium was changed to the induction medium, and the EBs were cultured for 2 days. About 30 *μ*L of Matrigel was used to embed a single EB. After Matrigel embedding, the EBs were cultured in an ultralow attachment 24-well plate (Corning) in expansion medium for 3 days. From day 10, the Matrigel-embedded EBs were cultured in the maturation medium in a 6 cm dish on an orbital shaker with a shaking speed of 65 rpm. On day 17, 10 ng/mL of BDNF and 10 ng/mL NT-3 were supplied to the maturation medium with a full-medium change every 3-4 days.

### 4.4. Animal and Surgical Procedures

Female Sprague-Dawley (SD) rats at the age of 6-8 weeks were used and maintained with an artificial 12 : 12-hour light-dark cycle. All animals received the subcutaneous injection of cyclosporine A (MCE) at the dose of 1 mg/100 g/day for three days before the operation. The drug was continuously applied during the whole experimental process. As shown in Figure S4, the animals were anesthetized by inhaling isoflurane (RWD Life Science) through a Mice&Rat Animal Anesthesia Machine (RWD Life Science) and kept warm on a heating pad. After the rats became fully unconscious, the dorsal hair was shaved and laminectomy was performed to expose the thoracic 10 (T10) level of the spinal cord. Complete transections were made, and a length of 4-5 mm of the spinal cord tissue was removed. The excessive blood in the cavity of the transected spinal cord tissue was cleaned with aseptic cotton, to ensure no spinal cord tissue was remaining by visual examination of the ventral side of the vertebrae of the spinal cord canal. For bead implantation, the beads in DMEM/F12 were aspirated into a pulled glass pipette. After the beads sank and piled at the tip of a vertically placed pipette, the beads were gradually relived into the cavity. A spatula was used to transfer the bulk gel and cerebral organoid to the SCI site. As for the liquid Matrigel injection, the Matrigel was injected with a prechilled pipette tip until the cavity was filled. A piece of subcutaneous adipose tissue was cut off and coved implants. Then, the incision was sutured, and a tail intravenous injection of 200 *μ*L of glucose solution was performed. 5,000 units/100 g/day of penicillin was injected for 3 days. The animals received the postsurgical care of manual emiction, which was performed until the bladder control reflex was reestablished. All the animal procedures were approved by the Animal Experimentation Ethics Committee at Tsinghua Shenzhen International Graduate School.

### 4.5. Tissue Processing and Immunofluorescent Staining

To harvest the spinal cord tissue, the rats were anesthetized with the isoflurane and placed in a supine position. An incision of about 3-4 cm was made at the chest to expose the heart. Each rat was perfused with 100 mL saline and 100 mL 4% PFA. The spine vertebrae were cut, and the dorsal parts were removed. The spinal cord was isolated and rinsed in PBS twice and then fixed in 4% PFA at 4°C overnight. After removing the PFA by washing with PBS, the samples were sequentially immersed with 10%, 20%, and 30% sucrose solutions and embedded into Optimal Cutting Temperature (OCT) compound (Tissue-Tek) and incubated overnight at 4°C. The tissue samples were frozen, and 30 *μ*m of slices was produced by cryosection. The same procedures of fixation, dehydration, and cryosection were applied for sectioning the cerebral organoids. For immunocytochemistry (ICC) staining, the cells were washed with PBS and fixed in 4% PFA for 10 minutes at room temperature. All the sectioned slices and cell samples were incubated with 10% normal donkey serum containing 0.5% and 0.1% Triton X-100 (Sigma), respectively. The primary antibodies (TuJ1, Abcam, ab18207, and BioLegend, 801201; GFAP, Sigma-Aldrich, G9269, and Abcam, ab4674; MAP2, Abcam, ab92434 and ab5392; Neurofilament Heavy (NF), Millipore, MAB1273; Iba1, Wako, 019-19741; Nestin, STEMCELL Technologies, 60091; OCT3/4, STEMCELL Technologies, 60093; PAX6, STEMCELL Technologies, 60094; SOX1, STEMCELL Technologies, 60095; human nuclear antigen (hNu), Abcam, ab191181; CD31, R&D System, AF3628; TBR1, Abcam, ab31940; TBR2, Millipore, AB15894; CTIP2, Abcam, ab18465; SATB2, Abcam, ab34735; GFP, Abcam, ab13970; PKC *ζ*, Santa Cruz, sc-17781; phosphorylated Vimentin (Ser55) (P-VIM), MBL, D076-3; Laminin, Abcam, ab11575; SOX2, Santa Cruz, sc-365823; phospho-histone H3 (Ser10) (pHH3), ZenBio, 301271) were incubated with the sample at 4°C overnight and followed by incubation of the secondary antibodies. The slices and cells were mounted with glycerol with DAPI (Sigma) and observed under a confocal microscope (Nikon).

### 4.6. Quantification and Statistical Analysis

Statistical analysis of all experiments was performed using GraphPad Prism software. The immunofluorescent images were processed by ImageJ software. For quantifying the axonal regeneration, an area of 0.407 mm^2^ was used for each image and three images were randomly captured at the rostral interface of the graft and the host tissues (1), the rostral part of the graft (2), the caudal part of the graft (3), and the caudal interface of the graft and the host tissues (4) (Figure 6(d)). Since the dying neurons/axons also emitted fluorescent signals and exhibited shapes of large dots together with the functional axons with fibrous structure, the intensity of dot signals was measured by employing “Erode” and “Dilate” of the original images, which were then subtracted from the measure of the whole image. Thereby, the intensity of fibrous axons was calculated. The cell counter of the ImageJ was used to count the Iba1-positive cells. Since all the comparable groups have the same sample size, paired two-tailed Student's *t*-test was used when analyzing the immunostaining results. The locomotor performance was assessed by the BBB score in the open-field test [72]. The scores were obtained from the observations of at least 3 rats. Unpaired Student's *t*-tests were used to compare differences between the two groups. All data were presented as means ± SEM. A *p* value less than 0.05 was considered statistically significant and marked by ∗, &, and #.

## Data Availability

The data that support the findings of this study are available from the corresponding author upon reasonable request.

## Supplementary Materials

Supplementary Materials Supplementary Figure 1: the diameters of collagen beads and Matrigel beads. Supplementary Figure 2: generation of neural stem cells from human iPSCs. Supplementary Figure 3: the live and dead staining of the cell-laden Matrigel beads and cell-laden bulk collagen. Supplementary Figure 4: establishment of complete transection injury of the spinal cord. Supplementary Figure 5: schematic of the procedure of implantation surgery and the spinal cord tissue after transplantation. Supplementary Figure 6: the immunostaining images of the transected spinal cords received collagen-DNA (col-DNA) hydrogel implantation groups in the forms of microbeads and bulk. Supplementary Figure 7: the quantification of axon regeneration in all groups. Supplementary Figure 8: the immunostaining images and quantification of CD31 in the grafts of the groups of cell-laden col-DNA microbeads and bulk collagen without cells.
