# Supplementary material for: CNS Organoid Surpasses Cell-Laden Microgel Assembly to Promote Spinal Cord Injury Repair
Source: Research (Wash D C). 2022 Aug 3;2022:9832128. doi: 10.34133/2022/9832128 (PMC9394056; doi:10.34133/2022/9832128)
Supplement: Supplementary Materials — Supplementary Figure 1: the diameters of collagen beads and Matrigel beads. Supplementary Figure 2: generation of neural stem cells from human iPSCs. Supplementary Figure 3: the live and dead staining of the cell-laden Matrigel beads and cell-laden bulk collagen. Supplementary Figure 4: establishment of complete transection injury of the spinal cord. Supplementary Figure 5: schematic of the procedure of implantation surgery and the spinal cord tissue after transplantation. Supplementary Figure 6: the immunostaining images of the transected spinal cords received collagen-DNA (col-DNA) hydrogel implantation groups in the forms of microbeads and bulk. Supplementary Figure 7: the quantification of axon regeneration in all groups. Supplementary Figure 8: the immunostaining images and quantification of CD31 in the grafts of the groups of cell-laden col-DNA microbeads and bulk collagen without cells. [file 9832128.f1.pdf]

Supplementary figures

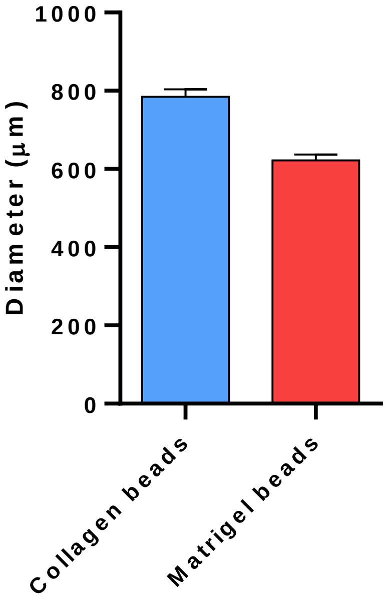

Supplementary Figure 1. The diameters of collagen beads and Matrigel beads.

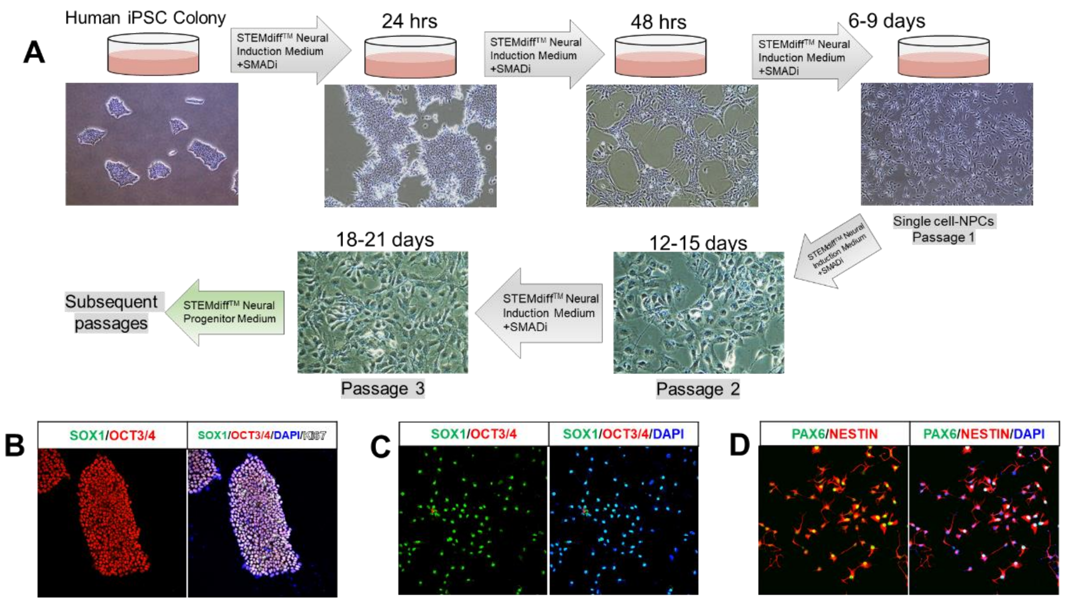

Supplementary Figure 2. Generation of neural stem cells from human iPSCs.

(A) The procedure to generate human neural stem cells from human iPSCs.

(B) Confocal micrographs show the iPSC colony expresses the marker of pluripotent stem cell OCT3/4, but not the marker of neural stem cells SOX1.

(C-D) After the neural induction process, almost all the cells are positive for SOX1 (C) and the neural progenitor markers PAX6 and Nestin (D).

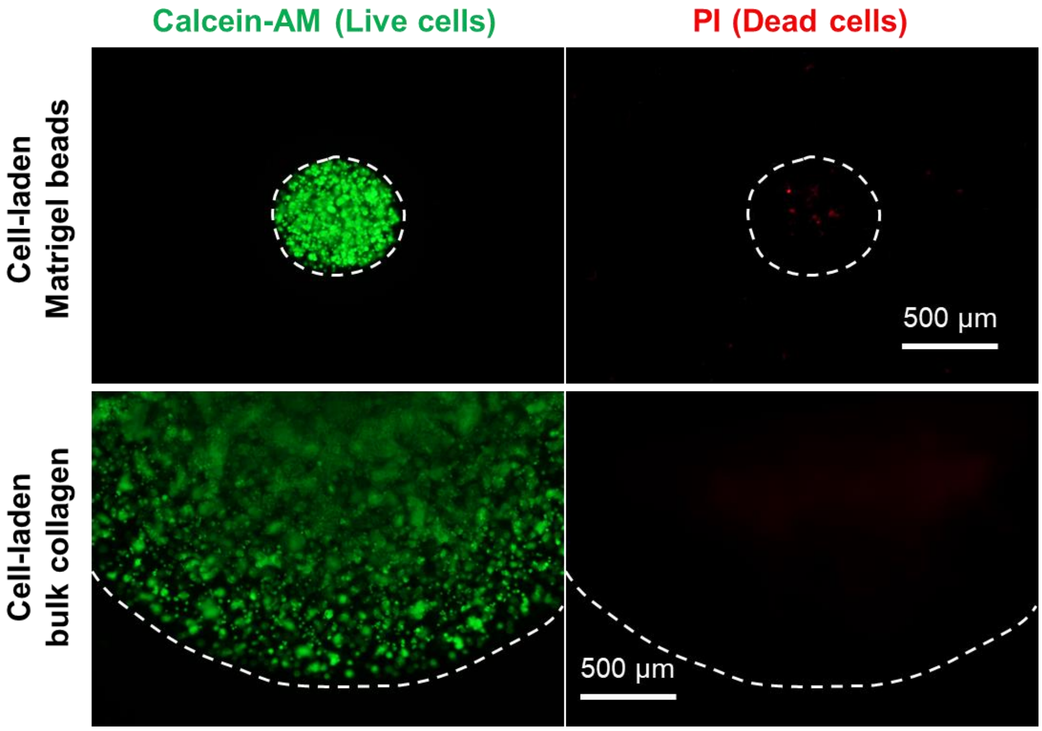

Supplementary Figure 3. The live & dead staining of the cell-laden Matrigel beads and cell-laden bulk collagen.

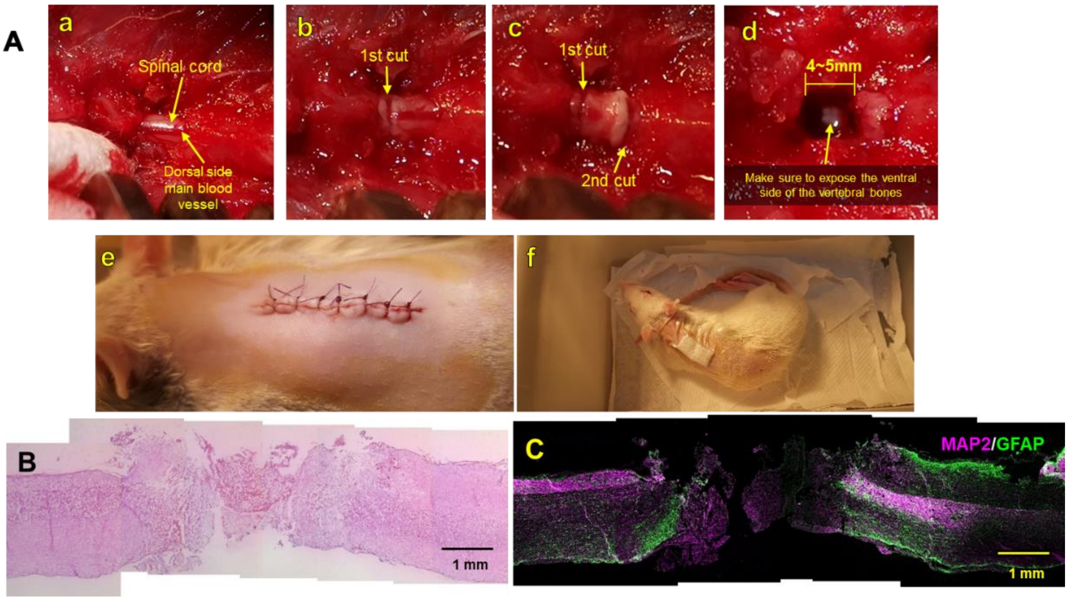

Supplementary Figure 4. Establishment of complete transection injury of the spinal cord. (A) The spinal cord was exposed after removing the spinous process of the vertebral bone of the T10 spine (a). Two complete cuts were made by using a microblade (b and c). A length of 4~5 mm of the spinal cord tissue was removed (d). The wound of the rat was sutured (e) and dressed (f).

(B) Ten days after the complete SCI was made, the spinal cord was harvested and cryosectioned. H&E staining shows the complete injury of the spinal cord.

(C) 10 days after the complete SCI was made, Immunostaining of the neuron-specific cytoskeletal proteins MAP2 and the astrocyte marker GFAP shows the complete transection of the spinal cord 10 days after the SCI transection.

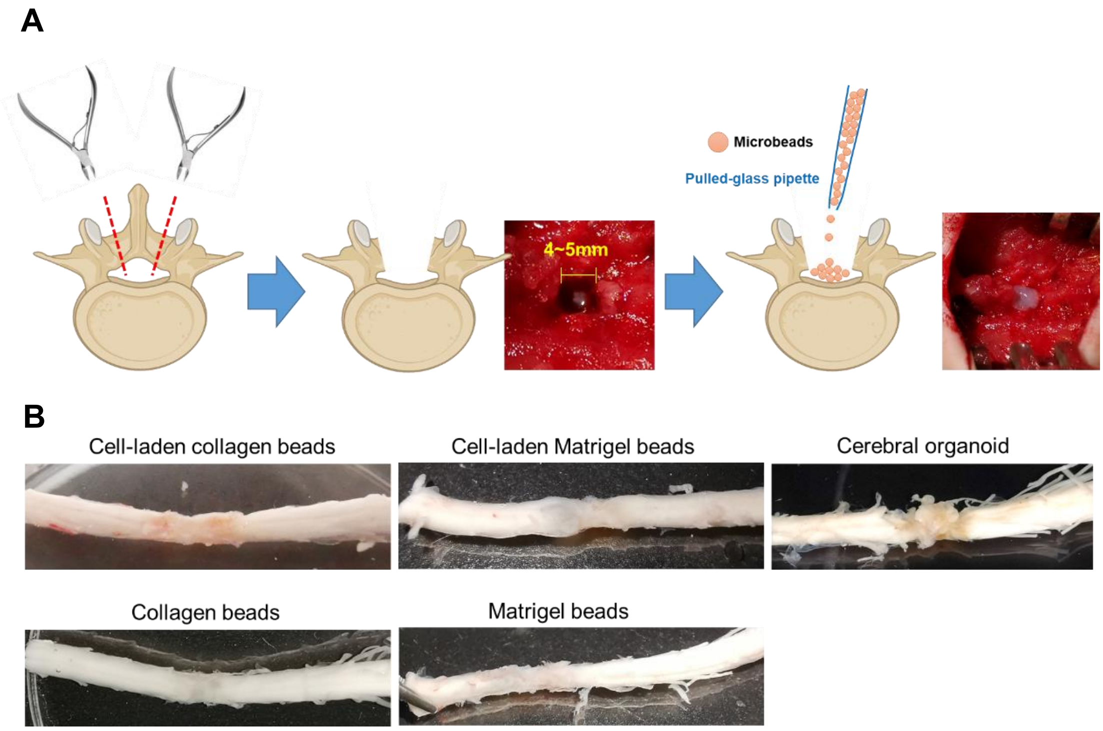

Supplementary Figure 5. Schematic of the procedure of implantation surgery and the spinal cord tissue after transplantation. The spinous process was cut and removed when performing laminectomy, and then the hydrogel beads were injected and filled the cavity after a section of spinal cord tissue was removed (A). The harvested spinal cord tissue in different groups at 2 months post-implantation (B).

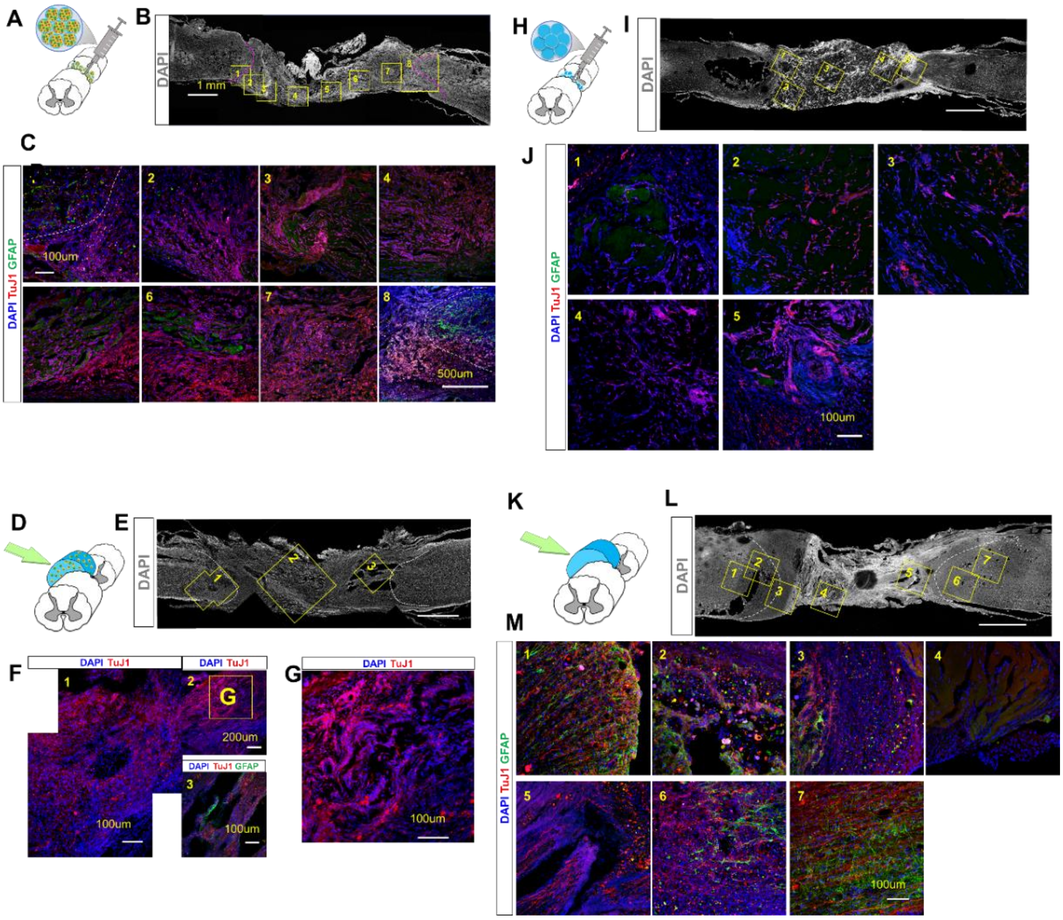

Supplementary Figure 6. The immunostaining images of the transected spinal cords received collagen-DNA (col-DNA) hydrogel implantation groups in the forms of microbeads and bulk.

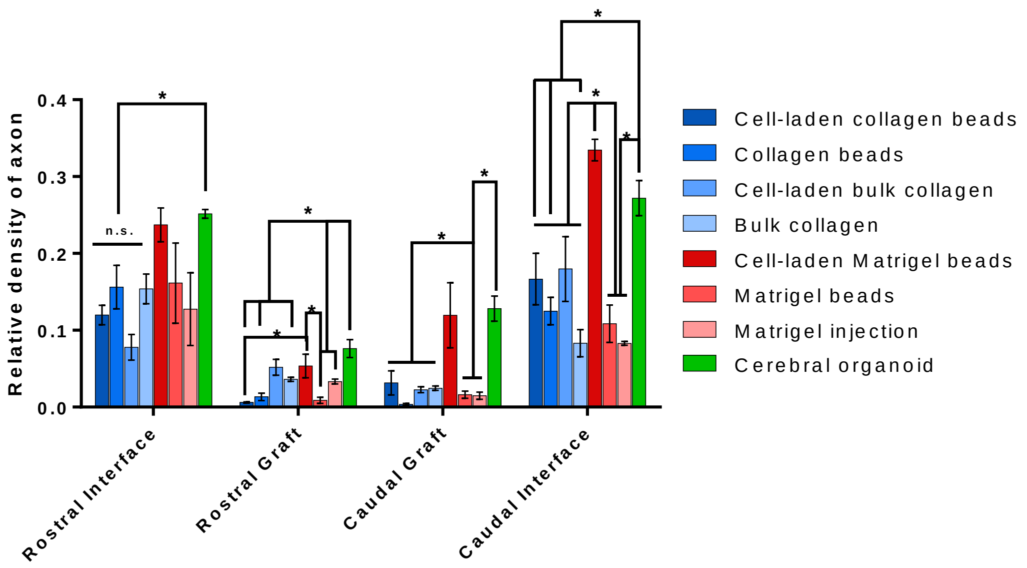

Supplementary Figure 7. The quantification of axon regeneration in all groups.

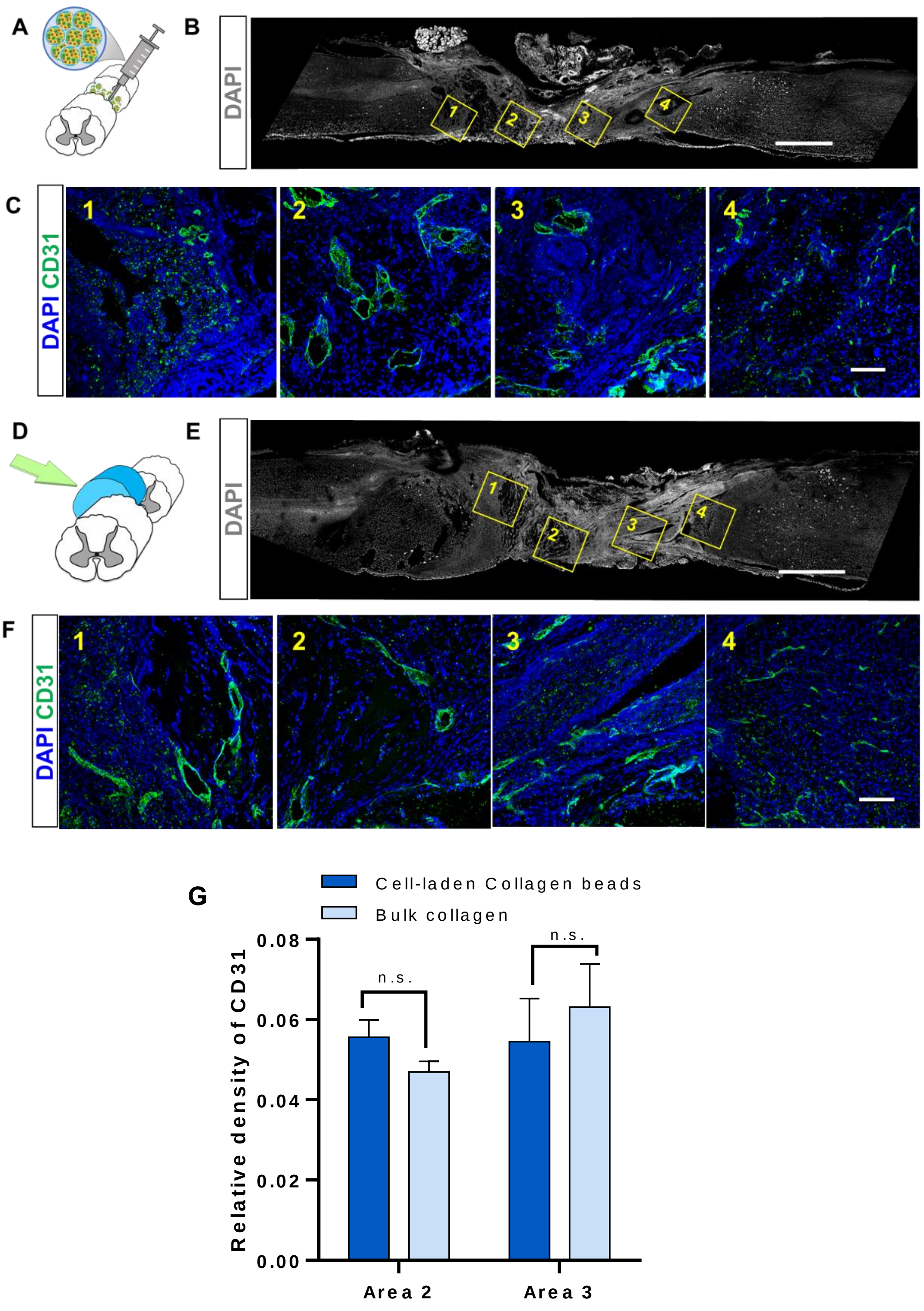

Supplementary Figure 8. The immunostaining images and quantification of CD31 in the grafts of the groups of cell-laden col-DNA microbeads and bulk collagen without cells. Scale bars, 100 μm (C and F); 1 mm (B and E)
